# Digital Application for Promoting Evidence-Based Children’s Oral Health to Control Early Childhood Caries: Randomized Control Trial on Parental Acceptance and Efficacy

**DOI:** 10.3390/jcm12072680

**Published:** 2023-04-04

**Authors:** Jameela Abdul Haq, Christian H. Splieth, Mhd Said Mourad, Annina Vielhauer, Ruba Abdulrahim, Manasi R. Khole, Ruth M. Santamaría

**Affiliations:** 1 Department of Preventive and Pediatric Dentistry, University of Greifswald, 17489 Greifswald, Germanyruba.abdulrahim@stud.uni-greifswald.de (R.A.);; 2 Department of Orthodontics, University of Greifswald, 17489 Greifswald, Germany

**Keywords:** ECC, digital apps, acceptance, efficacy

## Abstract

Background: Early childhood caries (ECC) remains a major global health problem. Various measures to prevent it have been implemented in the past, including those using digital applications. Aim: To evaluate the acceptance and efficacy of a digital application (FU-APP) based on evidence-based caries control recommendations for parents of children aged 6–72 months. Methods: Part 1, prospective questionnaire-based survey to test FU-APP (usage, acceptance, content information, usefulness, and satisfaction) filled out by parents (n = 22); Part 2, two-armed (test n = 20; control n = 23) care-based, randomized controlled trial, where the test arm received instructions verbally and via FU-APP, and the control arm received them only verbally. At baseline and follow-up (4 weeks), intraoral clinical indices (plaque index-API and caries-dmft) were recorded. Results: FU-APP was considered by parents to be a suitable tool for gaining knowledge about oral health practices for their children (all criteria >86%). No differences in the dmft levels were expected. However, API was significantly better at the follow-up in the test-arm (*p* = 0.01), with no differences in the control-arm (*p* = 0.72). Conclusion: A digital application can serve as an innovative tool to promote evidence-based oral hygiene recommendations among parents of children to control ECC. Its long-term usability and functionality should be tested.

## 1. Introduction

Early childhood caries (ECC), universally known as a complex, multifactorial and chronic disease, refers to a condition of the presence of one or more decayed, missing or filled tooth surfaces in any primary tooth in a child younger than 71 months of age [1]. Although ECC is preventable by nature, it continues to be the most common chronic disease in children worldwide [2], and its prevalence exhibits a wide variation range globally [3]. In Germany, the main caries experience at the defect level in the form of decayed, missing and filled teeth (dmft/DMFT) is concentrated in 14% of 3-year-olds, with 74% of untreated lesions [4]. This demands that suitable preventive oral hygiene measures be taken for caries control at the earliest stage possible [4]. Lack of correct oral health knowledge among parents can be one of the leading causes of ECC [5]. Parents play a central role in the early caries prevention process, since most preschool children are not capable of brushing their teeth correctly and cannot understand the importance of maintaining good oral health [6]. 

Multiple preventive strategies and interdisciplinary approaches have been implemented worldwide [7,8]. Specifically in Germany [7,9], one of the most promising concepts implemented for ECC prevention in the public insurance system includes early dental check-ups right from the eruption of the first primary tooth (FU1a-c), as well as risk-independent fluoride varnish application (FLA) and practical training in tooth-brushing with the caregiver (FUPr). These new activities are currently included in the National Health catalogue and are financially reimbursed [7]. 

It is known that digital technology might play an important role in oral health promotion in the contemporary world by providing interactive and user-friendly information to parents [10]. Multiple digital oral health promotion applications are available in the market for various purposes such as toothbrushing, exploring oral health status, brushing timers, appointment reminders, and targeting education of under-served population in developing countries, etc. [11,12,13,14]. In the literature, mostly the usability, acceptability, quality, and terminology adaptation of various oral health promotion applications have been discussed [15,16]. However, designing and testing a digital application providing evidence-based oral health knowledge in the population-specific language (German), specifically for the parents of younger children, to avoid ECC is rare [16]. Therefore, this study aimed to evaluate the acceptance and short-term efficacy of a digital application in improving evidence-based oral hygiene knowledge according to the current country recommendations among parents of very young children to control ECC. 

## 2. Materials and Methods

### 2.1. Ethical Approval

Ethical clearance was obtained from the Research Ethics Committee of Greifswald University, Germany (BB 97/20; trial registration No. NCT05515510). The study was conducted in accordance with the principles for medical research involving human subjects described by the Helsinki Declaration. 

### 2.2. Study Design

This study is split into two parts. Part 1 (also used as a pilot study) was a prospective questionnaire-based survey aimed to test the digital application “FU-APP”, developed to promote evidence-based oral hygiene knowledge among parents of young children to control ECC. 

**Figure 1:**
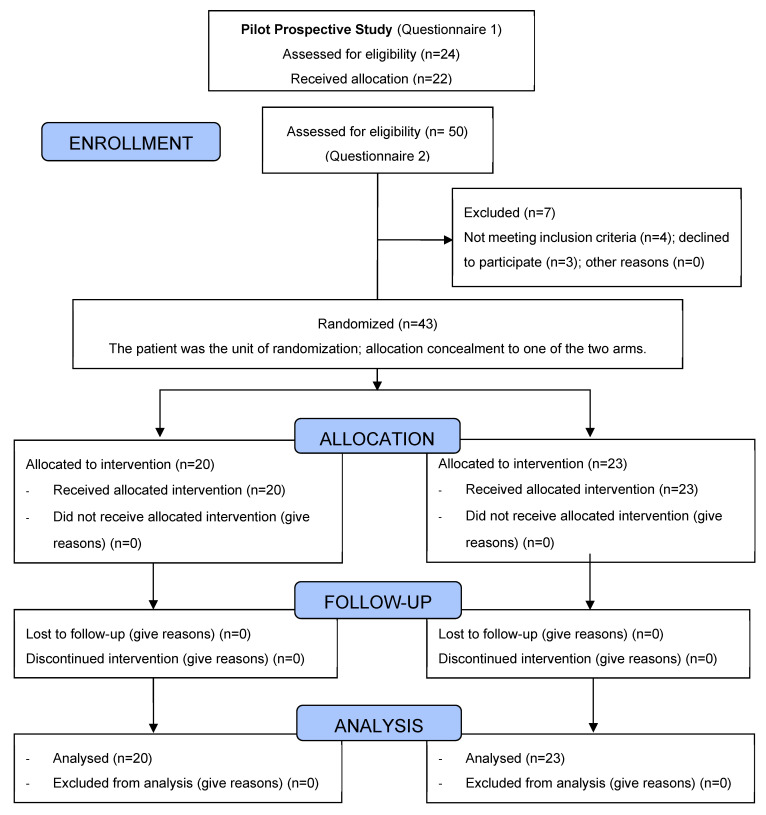
RCT Study CONSORT Diagram.

Part 2 corresponded to a two-armed (test and control) randomized controlled clinical trial, care-based study, which used intraoral clinical indices to measure the oral hygiene of participating children. Additionally, we assessed the acceptance and efficacy of FU-APP in improving parental knowledge, behavior, and self-reported practices/attitudes related to oral health preventive measures in the form of a questionnaire (Figure 1).

We included parents/children who could fully understand the German language; parents of medically fit children aged 6 to 72 months attending a specialized University Pediatric Dentistry Department for an oral health check-up. Parents/children who visit the clinic for an emergency treatment or pain were excluded.

### 2.3. Sample Size Calculation

To calculate the sample size, we used the comparison of proportions of two independent groups, as we expected to have many different endpoints/outcome variables; assuming a difference of 0.5 between groups at follow-ups, setting α = 5%, and power (1 − ß) = 0.80 resulted in 18 children for every group. Adding an expected drop-out of 10% led to a total sample size of 40 parents and 20 children being needed in both groups (test and control).

### 2.4. Application Development

FU-APP (from German: Frueherkennungsuntersuchung-App is a web-based application based on the current guidelines and oral health recommendations provided by the German counsel book, “Ratgeber 2020” [7]. It provides information related to oral health care to parents of children between 6 and 72 months of age. An account was made on a certified online web-development platform, and the application was developed on it between January and April 2021 in different phases. Initially, the consistency and understanding of FU-APP content was finalized. The language was simplified to make the dental terminology and recommendations comprehensive for users. In the following phase, photos, and videos to be included were captured and defined to make it interactive. The photos and videos were focused on plaque disclosing activity, correct brushing technique, amount of toothpaste to be used by parents according to age, and professional fluoride application. Thereafter, FU-APP was programmed and tested multiple times for technical errors, flow, and understanding. 

### 2.5. Questionnaires and Data Sheets

Part 1: Questionnaire 1 was designed to check FU-APP usage, acceptance, content information, usefulness, and satisfaction. 

The questions under the category “usage” focused on ease of understanding the FU-APP use, ease of navigation, and duration of use. Under the category “acceptance”, parents were asked if FU-APP provides an interactive insight to oral health. For the “content and information” category, parents were asked if the content was clear and easy to follow. In the next category, “usefulness”, they were asked if FU-APP can be useful to prevent ECC and whether the parents found the information useful to improve their knowledge and modify their behavior. The last section was related to the overall satisfaction with the performance of FU-APP. Response options were given in the form of a 5-point-Likert-scale from “completely agree” to “completely disagree”. 

For the randomized controlled trial (RCT), Questionnaire 2 included questions related to demographic characteristics, level of parental education, brushing information, amount of toothpaste used, behavioral habits, nutrition, previous experience with digital applications, etc. Prior to the intervention, the questionnaires were pilot tested on a group of 10 parents of children of similar age and setting to ensure that they were understandable and acceptable to the target group. The pilot tests did not reveal any major weaknesses in the design of the questionnaires, and the participating parents did not report any major difficulties in answering the questions. For the most part, the entire format of the questionnaires remained unchanged, and no questions were removed or added. A few questions were slightly revised to make the meaning clearer, and in only one case was the order of the questions changed to better reflect the parents’ assessment of the application’s acceptance and possible obstacles. 

### 2.6. Intervention 

Part 1: After receiving the informed consent from the parents, FU-APP was provided to the participants on an iPad. Once the parents had gained experience using the application, they were asked to fill out Questionnaire 1. This took place between May and August 2021.

Part 2, RCT: After receiving the informed consent from the parents, a computer-generated random number list with allocation concealment was used to assign children to 1 of the 2 arms. The randomization unit was the patient. Part 2 took place between October and December 2021.

The child was seated on the dental chair for a normal check-up, and the parents received the intervention according to the assigned arm in the form of oral/digital information. The treating dentist was blinded throughout the procedure until parents in arm 1 were asked to use FU-APP. The information provided to participants was based on the oral hygiene recommendations and current German guidelines (counseling in diet, brushing instructions, fluoride varnish application, nutrition, and behavioral habits) according to the age of the child [7]. Clinically, oral hygiene indices like Papillary Bleeding Index (PBI), Approximal Plaque Index (API), and caries indices (dmft/s) of the participating child were recorded. Parents in the test arm received additional digital information with the help of FU-APP during the dental visit.

Immediately after the appointment, all participant parents were asked to fill out Questionnaire 2. For both arms, the follow-up visit took place anytime between 2 and 4 weeks from baseline, which consisted of the same sequence of steps for the given intervention but with modified questions to compare changes in oral health knowledge. In addition, clinical indices were again recorded.

### 2.7. Outcomes 

#### 2.7.1. Primary Outcomes

Part 1, Pilot study: Level of acceptance and parental perception (usage, content information, usefulness, and satisfaction) about FU-APP. 

Part 2, RCT: Level of acceptance of FU-APP by parents and change in knowledge, behavior, and self-reported practices/attitude related to oral health preventive measures. 

#### 2.7.2. Secondary Outcome and Assessment

Clinical oral hygiene indices: Papillary Bleeding Index (PBI), Approximal Plaque Index (API), and caries index (dmft/s). 

### 2.8. Statistical Analysis

Data was analyzed using IBM-SPSS for Windows (v. 23). For Part 1 data, Spearman’s correlation coefficient was applied to assess the strength of association between overall satisfaction and usage, acceptance, content, and usefulness of FU-APP. The Kruskal—Wallis analysis of variance and the non-parametric Mann—Whitney U test were applied to assess differences between FU category and overall satisfaction. 

For Part 2, RCT—Normality of data was checked for quantitative variables. Comparisons between the test and control were done using independent samples *t*-test, or Mann—Whitney U test for quantitative variables (according to the variable normality), while comparisons of qualitative variables were done using Chi-square and Fisher exact tests, with Monte Carlo correction whenever indicated. Comparison of the baseline and follow-up in each group was done using Wilcoxon signed rank and Mc-Nemar tests. Significance was inferred at *p*-value < 0.05. 

## 3. Results

### 3.1. Part 1: Acceptance, Usability, and Parental Perception of the Digital Application (FU-APP)

#### 3.1.1. Baseline Characteristics 

Of the total 22 participants (1:1 parents of female/male children distribution), the mean age of the children was 3.65 ± 1.91 years (41.95 ± 19.18 mo.). The children dmft ranged between (0 and 16), with a mean of 5.59 ± 5.22. Out of the 22 children, 6 (27.3%) children had dmft index= 0, and 16 (72.7%) had a dmft > 0. 

#### 3.1.2. Outcomes

1. Usage of FU-APP: All participants completely agreed that the FU-APP was easy to use (n = 22; 100%). Almost all (n = 19, 86.4%) completely agreed that the FU-APP was easy to understand, that they liked the usage of the FU-APP, and that the usage time of FU-APP was also reasonable.
2. Acceptance of FU-APP: Almost 91% of participants (n = 20) completely agreed that FU-APP provides interactive insight to oral health, whereas 4.5% (n = 1) partially agreed with this statement.
3. Content and information of FU-APP: Of the 22 participants, 90.9% (n = 20) completely agreed that the information in FU-APP was clear, understandable, and easy to follow, and 95.5% (n = 21) completely agreed that the navigation was uniform.
4. Usefulness of FU-APP: 90.9% of the participants (n = 20) completely agreed that FU-APP can be useful for their child’s caries prevention, 86.4% (n = 19) completely agreed that FU-APP was useful to improve their child’s knowledge of oral hygiene, and 90.9% (n = 20) considered FU-APP as a helpful motivational oral hygiene tool.
5. Satisfaction regarding FU-APP: About 90.9% (n = 20) completely agreed that they would use this application again and recommend it to others. In addition, 86.4% (n = 19) of participants were overall satisfied with FU-APP.
6. Relationship between outcomes and assessed variables: There was a strong positive correlation between several variables—usage (understandability r = 0.630, *p* = 0.002), acceptance (interactive insight r = 0.817, *p* = 0.001), content and information (information clear, understandable, easy to follow r = 0.817, *p* = 0.001), and usefulness (knowledge r = 0.643, *p* = 0.001, and motivation improvement r = 0.815, *p* = 0.001). Description of all correlations are presented in Table 1.

### 3.2. Part 2: Randomized Controlled Trial

#### 3.2.1. Baseline Characteristics 

Of the 43 total participants, 20 parents were randomly assigned to the test and 23 to the control arm. In total, 21 (48.8%) were parents of girls and 22 (51.2%; *p* > 0.05) of boys. The mean age of children was 3.07 ± 1.65 years (control: 2.75; test: 3.35, *p* > 0.05; Table 2).

The parental education level was divided into the father’s and mother’s education categories. It showed a similar distribution when educational level was compared in each category (*p* > 0.05; Table 2). 

#### 3.2.2. Reasons of the Dental Visit

Most dental visits were for regular oral check-ups (n = 36; 83.7%), followed by treatment of carious lesions (n = 6; 13.95) or other teeth/gum problems (n = 4; 9.3%).

#### 3.2.3. Knowledge Comparison about the Best Oral Hygiene Practices

The assessed variables focused on brushing habits, fluoride content, and nutritional and behavioral habits. Parents were asked at baseline and at follow-up. 

As compared to the baseline in both arms, the percentage of “no one is brushing the teeth” decreased from 2 to 0 in the test arm at the follow-up (Table 3) depicting that all the children/parents had started brushing after receiving the intervention. There was a slight increase observed in the test group at the follow up (n = 18, 90%) in terms of usage of fluoridated toothpaste while brushing as compared to the baseline in the test group (n = 17, 85%). 

The correct timing to start cleaning children’s teeth is crucial to prevent ECC. In the test group, two parents had not started cleaning children’s teeth at the baseline; however, this improved at the follow-up after receiving the intervention. 

The sequence of brushing techniques was also taken into consideration with most parents who, at baseline, mentioned different techniques for brushing their children’s teeth. This practice changed significantly in both arms at follow-up with all parents in the test group (*p* = 0.02) and most in the control group (82.6%); *p* = 0.03 mentioning using the COI brushing technique.

Knowledge about the right amount of fluoride content in the toothpaste to be used with children increased at the follow-up in both arms; however, significant differences as compared to baseline were only seen in the test arm (*p* = 0.001 versus *p* = 0.1; Table 4).

#### 3.2.4. Knowledge Comparison about Nutritional Behavioral Practices

When parents were questioned about “diet modifications” parents can do to avoid ECC, 75% of parents in the test arm and 82.6% in the control arm reported that less sugary drink consumption can be of help. This reported knowledge increased in the test groups after the intervention to 100%. Further reported knowledge about nutritional and habitual practices is presented in Table 5.

#### 3.2.5. Attitude and Motivation Comparison

Lastly the crucial theme was touched upon by questioning whether the parent participants would like to receive oral health recommendations only verbally by a dentist, by a digital application, or by an application and dentist both. No differences could be found in either of the arms when baseline vs. follow-up or test vs. control arms were compared. However, most parents in the test group felt motivated to take care of the oral hygiene of their child after using FU-APP (63.2%), and these positive outcomes even increased during the time (80%). No significant changes were observed in the control group (Table 6).

#### 3.2.6. Caries Index, API and PBI Baseline, and Follow-Up Comparison

The overall baseline children’s dmft was 3.09 ± 4.84 (control: 3.13 ± 4.67; test: 3.05 ± 5.28; *p* > 0.05) ranging between (0 to 18). Out of the 43 children, 23 (53.5%) children had dmft index = 0, and 20 (46.5%) had a dmft > 0. No differences in the dmft levels were either expected or observed in either of the arms when baseline and follow-up were compared. However, the mean API was significantly better at the follow-up in the test group (*p*= 0.01), while no significant differences in the control group were observed (*p*= 0.72; Table 7).

## 4. Discussion

We developed a population-adapted, evidence-based, digital oral hygiene app (FU-APP) and evaluated its acceptability and short-term effectiveness to improve children’s oral hygiene and knowledge of parents of very young children to control ECC. Main findings showed remarkable FU-APP acceptance, with all participants agreeing that it provides interactive insight to oral health and that the information was clear, comprehensible, easy to follow, and useful. In addition, there was improvement in parental knowledge regarding oral hygiene practices for caries control and better plaque scores, indicating that FU-APP is potentially not only innovative, but also an effective tool to be considered for prevention of ECC at an early age. 

The empirical results presented here should be considered in light of some limitations. Thus, the sample size was calculated based on the expected differences in the various endpoints/outcome variables when comparing the test and control arms, which allowed us to work with a relatively small population. However, this would not allow the detection of minor effects. Still, the study identified statistically significant differences in questionnaire responses between groups and in plaque scores. Therefore, FU-APP proved to be clinically relevant with changes in the plaque scores and clear differences in the knowledge of the caretakers. Future studies on the implementation of FU-APP should include a larger population and concentrate on the caries outcome. The present study gives a good basis for planning such a trial. 

Caries still affects a large proportion of children worldwide [3,17]. In Germany, the prevalence of carious lesions in 3-year-olds is 14% (dmft: 0.48), with a significant group of children (38%) who experience severe caries (dmft: 3.57), mirroring the fact that caries polarization occurs at an early age [4,9]. Ever since the current caries prevalence data in children was published, new caries control policies at the country level have been implemented. In mid-2019, caries-preventive strategies for primary dentition have been further expanded, including early dental visits right from the eruption of the first primary tooth [7]. In the light of new preventive strategies, this is an excellent opportunity to tackle parents with a digital application, overcoming possible social barriers, with an interactive way to approach parents and complement the traditional dental visit [7]. However, it must always be borne in mind that these individual caries-control strategies depend strongly on parental cooperation to follow tooth-brushing recommendations with their children, as well as to bring their children from early ages for dental check-ups, which can be particularly problematic in risk groups. 

Digital applications are used massively in the field of general medical health. A good example of these are the research-tested applications for breast cancer awareness and prevention [18]. After testing, it was discovered that such apps are suitable in behavioral therapy for people with different health literacy levels. However, these should be tailored for specific contexts (culture, race, and ethnic groups) [18]. The literature similarly supports that there is a requirement for culturally-tailored smartphone applications [18,19,20]. In this regard, the developed FU-APP might help to address the individual population needs and increase motivation [16]. The future vision of FU-APP also includes a customized version to reach other communities present in Germany, such as refugees or people whose primary language is not German. 

In our study, a very strong positive correlation was found between overall satisfaction and usefulness of FU-APP in motivation of the child regarding oral hygiene and adapting behavior (r = 0.815, *p* = 0.001; Table 1). The feeling of motivation is very subjective; however, our findings showed that oral hygiene–plaque scores indicated some level of improvement, with significant differences between the groups. 

Children are dependent on their caretakers for oral hygiene maintenance [21]. Thus, parents play a key role in the prevention of dental caries, as their oral health knowledge and aptitudes determine the oral health of their children [22,23,24]. However, this is highly dependent on factors like level of parental education, culture, beliefs, etc. [25,26] and varies greatly across the globe. It has been reported that parents with an academic degree had superior knowledge about better oral hygiene practices than parents with only school education [21,26,27,28]. These results are concurrent with our study where 50-65% mothers and 60–65% fathers had no university education. Their knowledge and self-reported attitude/practices, however, improved significantly after the study (test *p* = 0.02), especially regarding the right time to start brushing and the reported brushing technique. This was also evident considering that at the beginning of the study almost half (48.8%) of the parents did not know the right amount of fluoride content in toothpaste (control: 55%; test: 43.5%), but at follow-up most parents (80%) in the test group mentioned the right answer (*p* = 0.001), whereas no significant changes were observed in the control arm. In addition, different studies in literature have shown that, despite good levels of oral health literacy, there is still confusion among parents regarding the correct amount of fluoride to be used. Therefore, health promotion along with health education provided in an interactive way, e.g., through an app, might support a reduction of this issue [21,25,28,29]. Considering the strong association between caries decline and fluoride use [30], there is evidence showing that suboptimal fluoride concentration (<1000 ppm Fl) may negatively influence the caries levels [30]. In Germany, therefore, since 2018, the European fluoride recommendations (>1000 ppm Fl) have been adopted [31]. 

In the present study, no changes in caries levels were expected due to the short time frame between baseline and follow-up. However, there were already significant differences in plaque levels of children in the test group. It is known that biofilm accumulation as result of frequent carbohydrate exposure favors biofilm formation leading to caries development. Current strategies for caries control are focused on controlling the lesion activity, using minimally invasive approaches, such as biofilm control and fluoridation [32,33]. 

A variety of digital applications have been tested in multiple randomized clinical trials. One RCT conducted in the Netherlands evaluated the effectiveness of the WhiteTeeth mobile app in improving the oral hygiene of adolescent orthodontic patients. The results showed that at the 6-week follow-up, the intervention led to a significant decrease in gingival bleeding (−3.74), and an increase in the use of fluoride mouth rinse (+1.93), with significant differences to the control arm. It was concluded that, for optimal improvement in oral hygiene, the usual care should be combined with extra digital tools as an aid [34]. In our study, there has been no change in the caries levels, however the improvements in plaque score could mean per se a decrease in caries risk. 

Recently the Coronavirus Disease (COVID-19) pandemic posed many challenges. The concept of home office and digital work became more popular, and digital technology, largely using apps (contact tracing, preventive measures, case tracker, guidelines for travel and living, information related to quarantine, etc.) [35,36,37], took over in all the sectors of the professional world. Even in many areas of health care, it is intended to enable doctors (e.g., psychotherapists) to prescribe digital health applications to their patients, which can be reimbursed by statutory health insurance funds (§§ 33a and 139e Fünftes Buch Sozialgesetzbuch). FU-APP could also serve to provide parents with useful evidenced-based current recommendations in oral hygiene and prevention of the most common oral diseases (caries and gingivitis) in children [7] when face-to-face appointments are not doable. 

## 5. Conclusions

The concept of digital application specifically for parents of younger children to control ECC is very limited. The development of such an application has the potential to complement the existing methods of oral health education and promotion for caries prevention among parents right from a very young age of their children in a user-friendly manner. Our findings showed that FU-APP was, overall, well-accepted, and at the short-term, there was improvement in the knowledge of parents regarding oral hygiene practices for caries control, as well as in plaque score levels of their children. 

In conclusion, in this current modern age of digital dentistry—a population-adapted, evidence-based, digital oral hygiene app (FU-APP) can serve as an innovative and suitable tool to promote oral hygiene knowledge among parents of young children to control ECC. Further appraisal of its long-term usability and functionality, also in clinical terms, should be tested in larger populations; however, it is not expected that different results than the ones we presented will be seen. Nonetheless, this can further strengthen the concept of digital applications being actively used for oral health promotion.

## Figures and Tables

**Table 1:** Correlations between parental overall satisfaction and baseline variables.

Questionnaire Items	Overall Satisfaction	
**Usage**	**r**	*p* **-Value**
FU-APP was easy to understand?	0.630	0.002 *
I liked the usage of this FU-APP?	0.263	0.238
The usage time for this FU-APP was measurable/suitable?	0.210	0.348
**Acceptance**		
FU-APP provided interactive insight to oral health?	0.817	0.001 *
**Content and information**		
The information in FU-APP was clear and understandable and easy to follow?	0.817	0.001 *
Navigation was uniform?	0.087	0.702
**Usefulness**		
App can be useful for prevention of caries in my child?	0.313	0.156
App was useful to improve the knowledge about oral health hygiene of my child?	0.643	0.001 *
The app was helpful in motivating me about my child’s oral hygiene and adapting my behavior accordingly?	0.815	0.001 *

r = correlation coefficient; * statistically significant.

**Table 2:** Demographic profile of participants in the randomized clinical trial (Part 2).

Demographic Characteristics		Test (n = 20)	Control (n = 23)	*p*-Value
Age in years	Mean (SD) (a)	2.75 (1.97)	3.35 (1.30)	0.26
Age in months		36.10 (22.59)	44.13 (15.38)	0.19
Age category * (b)	6–9 months	2 (10%)	0 (0%)	PMC: 0.18
	10–20 months	5 (25%)	2 (8.7%)	
	21–33 months	2 (10%)	3 (13%)	
	34–72 months	11 (55%)	18 (78.3%)	
Child’s sex (b)	Female	10 (50%)	11 (47.8%)	0.89
	Male	10 (50%)	12 (52.2%)	
Parent’s sex (b)	Female	13 (65%)	15 (65.2%)	0.99
	Male	7 (35%)	8 (34.8%)	
Mother’s education (b)	No degree	0 (0%)	1 (4.3%)	PMC: 0.57
	Elementary school	0 (0%)	4 (17.4%)	
	Middle/secondary school	4 (20%)	2 (8.7%)	
	Vocational training	5 (25%)	7 (30.4%)	
	Technical college/diploma	1 (5%)	1 (4.3%)	
	Academic/university education	6 (30%)	5 (21.7%)	
	Higher education	4 (20%)	3 (13%)	
Father’s education (b)	No degree	0 (0%)	1 (4.3%)	PMC: 0.87
	Elementary school	1 (5%)	2 (8.7%)	
	Middle/secondary school	3 (15%)	2 (8.7%)	
	Vocational training	9 (45%)	9 (39.1%)	
	Technical college/diploma	0 (0%)	0 (0%)	
	Academic/university education	6 (30%)	5 (21.7%)	
	Higher education	1 (5%)	4 (17.4%)	

(a): Independent samples *t*-test, (b): chi-squared test; *: Months distribution according to current age distribution recommendations under the German Health System (REF); PMC: Monte Carlo corrected *p*-value.

**Table 3:** Child’s oral hygiene practices at baseline and follow-up in two study arms.

Oral Hygiene Practices		Test (n = 20)		Control (n = 23)		*p*-Value
		Baseline	Follow-Up	Baseline	Follow-Up	PMC
Who cleans the child’s teeth?	Child alone	2 (10%)	2 (10%)	8 (34.8%)	10 (43.5%)	Baseline 0.02 * Follow-up 0.02 *
	Parents alone	6 (30%)	6 (30%)	1 (4.3%)	1 (4.3%)	
	Child and parents	10 (50%)	12 (60%)	13 (56.5%)	11 (47.8%)	
	No one	2 (10%)	0 (0%)	0 (0%)	0 (0%)	
	Someone else (Nursery)	0 (0%)	0 (0%)	1 (4.3%)	1 (4.3%)	
	*p*-value	0.60		0.16		
With what do you brush your child’s teeth?	Toothbrush	2 (10%)	1 (5%)	2 (9.1%)	2 (8.7%)	Baseline 0.85 Follow-up 0.85
	Toothbrush and toothpaste without fluoride	1 (5%)	1 (5%)	3 (13.6%)	4 (17.4%)	
	Toothbrush and toothpaste with fluoride	17 (85%)	18 (90%)	17 (77.3%)	17 (73.9%)	
	*p*-value	0.41		0.66		
How often are your child’s teeth brushed?	1 time a day	7 (35%)	3 (15%)	7 (30.4%)	5 (21.7%)	Baseline 0.31 Follow-up 0.31
	2 or more times a day	11 (55%)	17 (85%)	16 (69.6%)	18 (78.3%)	
	Less than 1 time a week	2 (10%)	0 (0%)	0 (0%)	0 (0%)	
	*p*-value	0.86		0.32		

PMC: Monte Carlo corrected *p* value; * statistically significant at *p*-value < 0.05.

**Table 4:** Knowledge about the best oral hygiene practices at baseline and follow-up.

Knowledge about Best Oral Hygiene Practices		Test (n = 20)		Control (n = 23)		*p*-Value
		Baseline	Follow-Up	Baseline	Follow-Up	PMC
When should we begin with cleaning the teeth?	Less than 6 months	4 (20%)	7 (35%)	5 (21.7%)	5 (21.7%)	Baseline 0.42 Follow-up 0.053
	6–12 months	11 (55%)	13 (65%)	10 (43.5%)	11 (47.8%)	
	12–24 months	2 (10%)	0 (0%)	5 (21.7%)	5 (21.7%)	
	>24 months	1 (5%)	0 (0%)	3 (13%)	2 (8.7%)	
	Not started yet	2 (10%)	0 (0%)	0 (0%)	0 (0%)	
	*p*-value	0.02 *		0.56		
What is the right order to brush?	COI brushing technique	14 (70%)	20 (100%)	14 (60.9%)	19 (82.6%)	Baseline 0.80 Follow-up 0.24
	Horizontal vertical	4 (20%)	0 (0%)	7 (30.4%)	3 (13%)	
	The direction does not matter	2 (10%)	0 (0%)	2 (7.7%)	1 (4.3%)	
	*p* value	0.02 *		0.03 *		
What should be the fluoride content? ^+^	No fluoride	0 (0%)	1 (5%)	0 (0%)	0 (0%)	Baseline 0.56 Follow-up 0.39
	500 ppm fluoride	1 (5%)	2 (10%)	2 (9.1%)	4 (17.4%)	
	1000 ppm fluoride	9 (45%)	16 (80%)	12 (54.5%)	14 (60.9%)	
	>than 1400 ppm fluoride	0 (0%)	0 (0%)	1 (4.5%)	1 (4.3%)	
	I don’t know	10 (50%)	1 (5%)	7 (31.8%)	1 (4.3%)	
	*p*-value	0.001 *		0.10		

PMC: Monte Carlo corrected *p*-value; * statistically significant at *p* < 0.05; COI (chewing, outside, and inner surfaces); ^+^ missing data corresponds to non-respondents.

**Table 5:** Knowledge about nutritional and habitual practices at baseline and follow-up.

Knowledge about Nutritional and Habitual Practices		Test (n = 20)		Control (n = 23)		*p*-Value
		Baseline	Follow-Up	Baseline	Follow-Up	PMC
By which nutrition modifications can we avoid ECC?	Decreasing sugary food/ beverages	15 (75%)	20 (100%)	19 (82.6%)	22 (95.7%)	Baseline 0.14 Follow-up 0.22
	Avoiding bottle feeding	5 (25%)	0 (0%)	1 (4.3%)	0 (0%)	
	Diet has no effect	0 (0%)	0 (0%)	2 (8.7%)	0 (0%)	
	I don’t know	0 (0%)	0 (0%)	1 (4.3%)	1 (4.3%)	
	*p*-value	0.23		0.43		
Which behavioral habits do you think can damage your child’s dentition?	Thumb sucking	7 (35%)	9 (45%)	15 (65.2%)	20 (86.9%)	Baseline 0.28 Follow-up 0.01 *
	Nail biting	0 (0%)	0 (0%)	0 (0%)	0 (0%)	
	Thumb sucking + nail biting	11 (55%)	11 (55%)	7 (30.4%)	2 (8.7%)	
	Behavioral habits have no effect	0 (0%)	0 (0%)	0 (0%)	1 (4.4%)	
	I don’t know	2 (10%)	0 (0%)	1 (4.4%)	0 (0%)	
	*p*-value	0.41		0.18		

PMC: Monte Carlo corrected *p*-value; * statistically significant

**Table 6:** Attitude and motivation at baseline and follow-up in the two study arms.

Attitude and Motivation		Test (n = 20)		Control (n = 23)		*p*-Value
		Baseline	Follow-Up	Baseline	Follow-Up	PMC
How would you have recommendations?	Only through the dentist	7 (35%)	6 (30%)	12 (52.2%)	15 (65.2%)	Baseline 0.22 Follow-up 0.06
	Via apps only	0 (0%)	0 (0%)	1 (4.3%)	1 (4.3%)	
	Through the dentist and digital apps	13 (65%)	12 (60%)	9 (39.1%)	6 (26.1%)	
	This makes no difference	0 (0%)	2 (10%)	1 (4.3%)	1 (4.5%)	
	*p*-value	0.64		0.28		
Do you feel this appointment influences your motivation to brush your children’s teeth? ^+^	Yes	12 (63.2%)	16 (80%)	0 (0%)	1 (6.7%)	Baseline <0.001 * Follow-up <0.001*
	No	2 (10.5%)	0 (0%)	15 (100%)	14 (93.3%)	
	It doesn’t make a difference	5 (26.3%)	4 (20%)	0 (0%)	0 (0%)	
	*p*-value	0.10		0.32		

PMC: Monte Carlo corrected *p*-value; * statistically significant at *p* < 0.05; ^+^ missing data corresponds to non-respondents.

**Table 7:** API and PBI indices at baseline and follow-up.

	Test (n = 20) Mean (SD)		Control (n = 23) Mean (SD)		Mann-Whitney U *p*-Value	
	Baseline	Follow-Up	Baseline	Follow-Up	Baseline	Follow-Up
API	29.00 (23.37)	20.50 (15.72)	30.22 (15.41)	33.91 (24.63)	0.32	0.03 *
	*p*-value: 0.01 *		*p*-value: 0.72			
PBI	1.00 (3.08)	0.50 (2.24)	0.44 (2.09)	0.87 (4.17)	0.47	0.95
	*p*-value: 0.32		*p*-value: 0.66			

API: Approximal Plaque Index; PBI: Papillary-Bleeding-Index; * statistically significant

## Data Availability

Not applicable.
